# Supplementary figures and images for: Repeated tDCS at clinically-relevant field intensity can boost concurrent motor learning in rats
Source: bioRxiv. 2025 Jan 16:2025.01.15.633248. Preprint. [Version 1] doi: 10.1101/2025.01.15.633248 (PMC11761702; doi:10.1101/2025.01.15.633248)

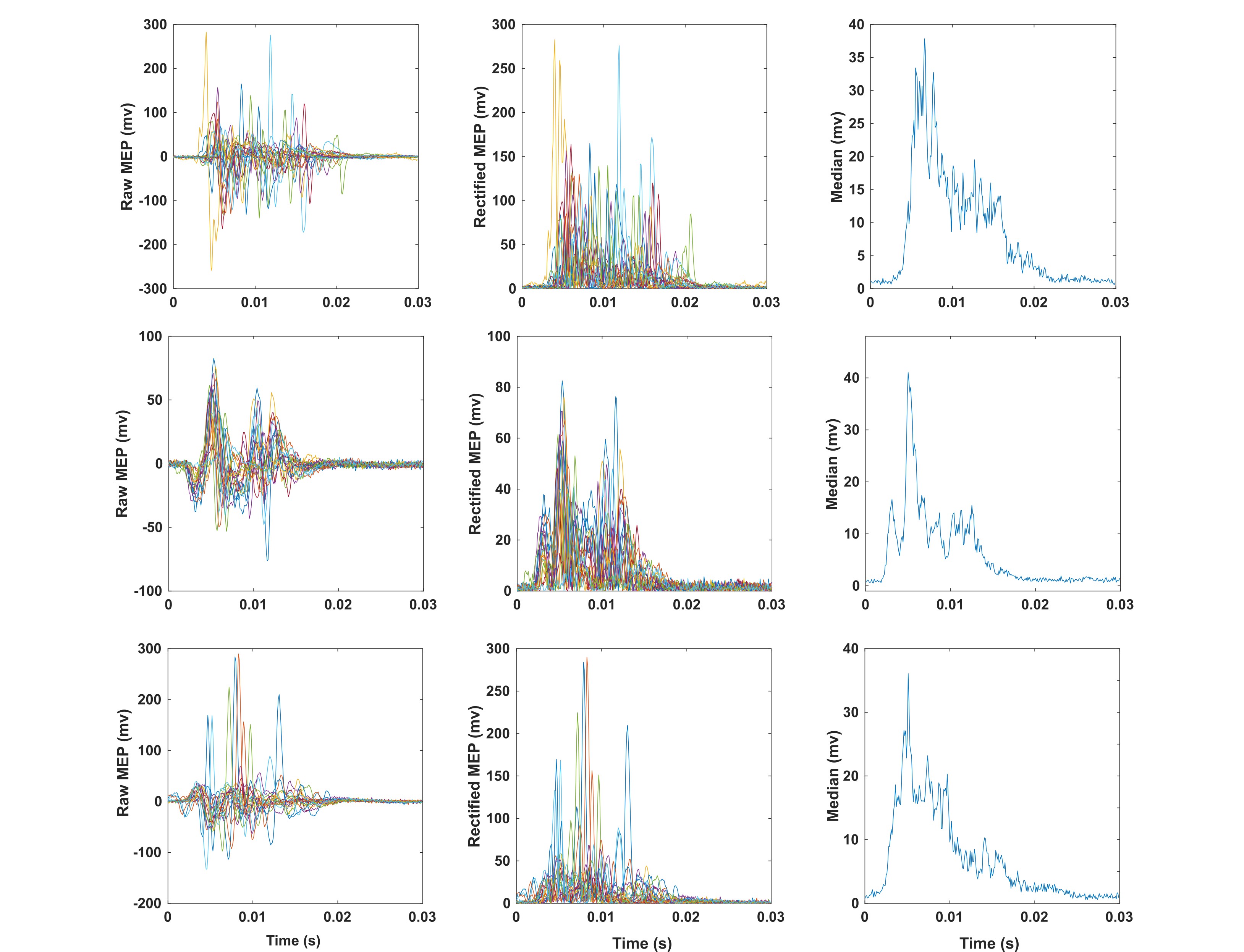

Supplement: Supplement 1 — Figure 4-1: Motor evoked potential (MEP) measurements. Left: Raw MEP signals (stimulation artifacts truncated) for 20 trials (color cycle) 10 ms after the last pulse of stimulation. Middle: rectified MEP. Right: median across repeats, here for the 140% condition stimulation. A/B/C are for three different animals. [file media-1.jpg]

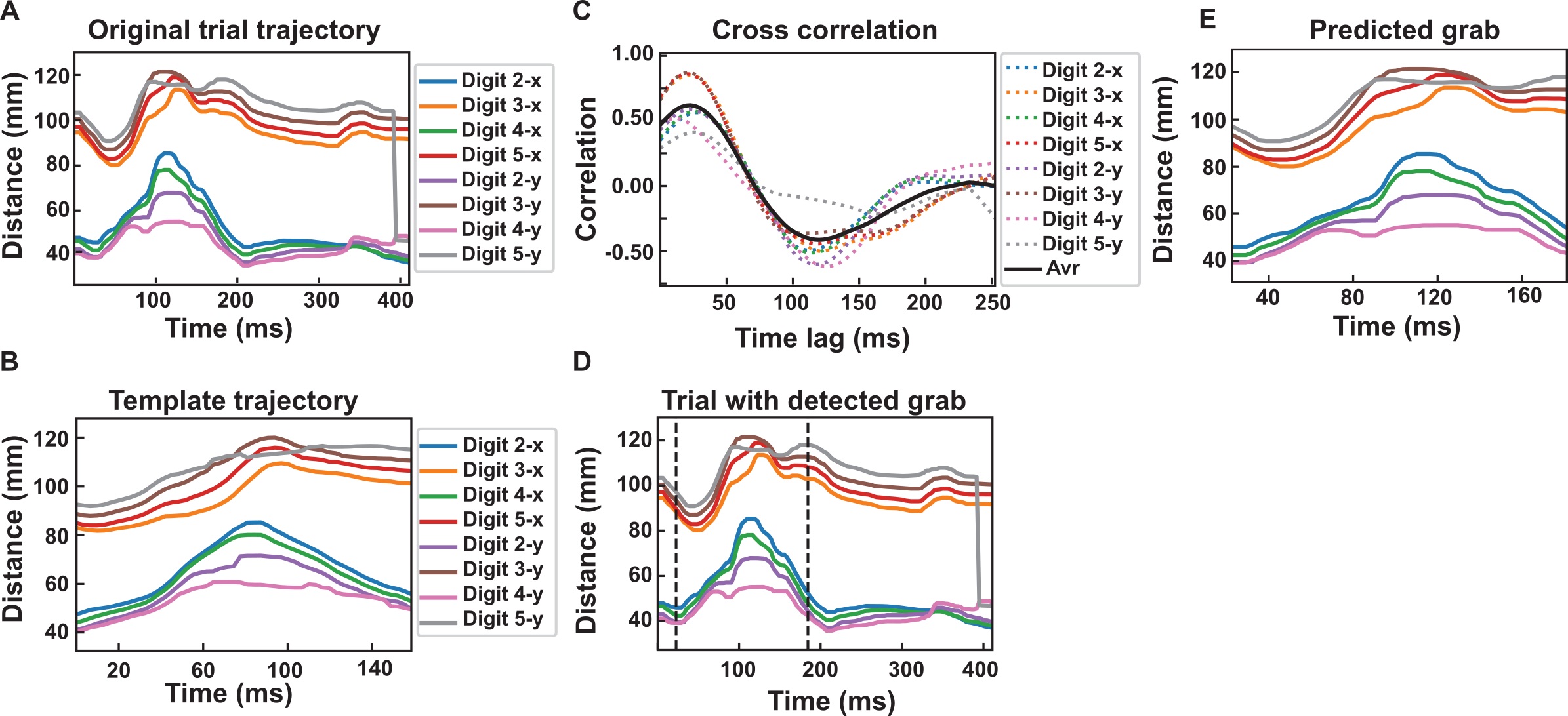

Supplement: Supplement 2 — Figure 5-2: Reaching trajectories for four digits. A: The trajectory data for a single grab video, covering the entire 400 ms duration. B: The template trajectory, derived from the average grab across three trials, specifically at the time of the grab (150 ms). C: The cross-correlation analyses were conducted for each digit compared to the template grab, with an average computed across all digits. The peak correlation is utilized to predict the location of the grab (30ms). D: The detected grab location shown in the original trial (30 ms – 180 ms). E: The predicted grab locations separately within the time window of 30 ms to 180 ms. [file media-2.jpg]

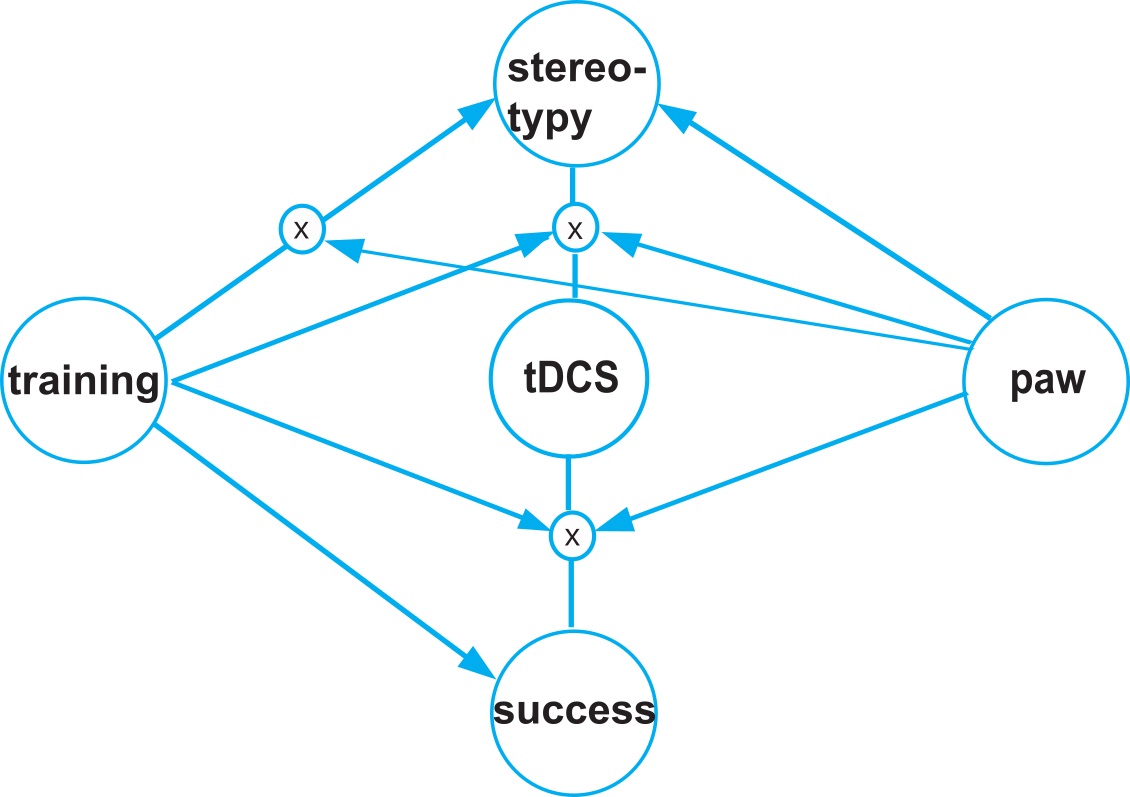

Supplement: Supplement 3 — Figure 5-1: Summary of statistical analysis including paw-preference as a factor. Links indicate significant regression parameters (p<0.05) in two separate linear mixed effect models, one for the number of successes and another for stereotypy as dependent variables. tDCS, training day and paw were the dependent variables (fixed effects), while animal was a random effect variable. Missing arrows indicate non-significant regression parameters (p>0.05). Not all possible 3-way interactions were tested. We caution that these post hoc analyses are not corrected for multiple comparisons and are not adequately powered. They should be considered exploratory and would need to be confirmed in new planned experiments. [file media-3.jpg]
